# Behind the Scenes: Nod-Like Receptor X1 Controls Inflammation and Metabolism

**DOI:** 10.3389/fcimb.2020.609812

**Published:** 2020-12-04

**Authors:** Tiia Snäkä, Nicolas Fasel

**Affiliations:** Department of Biochemistry, University of Lausanne, Epalinges, Switzerland

**Keywords:** nod-like receptor X1, infection, inflammation, mitochondria, metabolism

## Abstract

Regulatory Nod-like receptors (NLRs) are a subgroup of the cytosolic NLR family of pathogen recognition receptors (PRRs). These receptors can tune the innate immune responses triggered by the activation of other PRRs by either augmenting or attenuating the activated pro-inflammatory signaling cascades. Nod-like receptor X1 (NLRX1) is the only known mitochondria-associated negative regulatory NLR. NLRX1 attenuates several inflammatory pathways and modulates cellular processes such as autophagy and mitochondrial function following infection or injury. Using both *in vitro* expression and *in vivo* experimental models, NLRX1 is extensively described in the context of anti-viral signaling and host-defense against invading pathogens. More recently, NLRX1 has also gained interest in the field of cancer and metabolism where NLRX1 functions to attenuate overzealous inflammation in various inflammatory and autoimmune diseases. However, the exact function of this novel receptor is still under debate and many, often contradictory, mechanisms of action together with cellular localizations have been proposed. Thus, a better understanding of the underlying mechanism is crucial for future research and development of novel therapeutical approaches. Here, we summarize the current findings on NLRX1 and discuss its role in both infectious and inflammatory context.

## Introduction

To respond to infection, innate immune cells of the host express multiple pathogen recognition receptors (PRRs) that recognize different danger- and pathogen associated molecular patterns (DAMPs and PAMPs) and induce the expression of key proinflammatory pathways. This array of PRRs include the membrane-bound Toll-like receptors (TLRs) and C-type lectin receptors (CLRs) and the cytosolic Rig-I-like helicase receptors (RLRs) and nucleotide-binding oligomerization domain (NOD)-like receptors (NLRs). While the role of TLRs, CLRs, and RLRs in pathogen recognition and cellular defense is relatively well defined, the function and mechanism of action of most NLRs is less understood.

The NLR family contains 22 distinct members in humans and 34 in mice. These members can be divided into inflammasome- and non-inflammasome forming subgroups (Franchi et al., 2009). NLRs are composed of three domains: a leucine-rich repeat (LRR) containing C-terminal domain for ligand sensing, a central conserved nucleotide-binding domain (NACHT) necessary for oligomerization upon activation, and a variable N-terminal effector domain to recruit signaling adaptors. Upon activation, by interacting with downstream factors, the N-terminal domain will mediate the upregulation of the downstream signaling pathways, which are involved in different cellular processes including inflammation and apoptosis (Franchi et al., 2009). Many NLRs have clear pathogen-recognition functions and directly recognize pathogen derived molecules. For example, NOD1 and NOD2, the first described NLRs, are directly involved in bacterial sensing by recognizing bacterial peptidoglycan (Keestra-Gounder and Tsolis, 2017). The best characterized and most studied members of the NLR family are the inflammasome forming NLRs such as NLRP3 (NOD-, LRR- and pyrin domain-containing protein 3). The latter play a role in the immune response against multiple pathogens including viruses, bacteria and protozoan parasites (Kim and Jo, 2013; Lupfer and Kanneganti, 2013; De Carvalho and Zamboni, 2020). While the role of NLRs in antiviral and -bacterial signaling is relatively well defined, their function and mechanism of action in parasitic disease is less understood.

Certain NLRs, however, also function in processes independent of infection. These latter receptors act as cytosolic innate sensors to different DAMPs and induce an inflammatory response to cellular damage or stress (Kufer and Sansonetti, 2011; Mason et al., 2012). Furthermore, NLRs play important roles in many inflammatory diseases and metabolic disorders where inflammation is a key mediator of disease progression. For example, the NLRP3 inflammasome have been shown to be involved beyond pathogen recognition in driving inflammation in different autoimmune disorders and in tumorigenesis (Martinon et al., 2002; Kufer and Sansonetti, 2011; Allen, 2014). There is growing evidence, however, that some NLRs function independently of inflammasome formation and may even negatively regulate inflammation (Allen, 2014; Coutermarsh-Ott et al., 2016a). This recent subgroup of negative regulatory NLRs is composed of three members, NLRP12, NLRC3, and NLRX1. These receptors function to attenuate diverse signaling pathways such as the nuclear factor-_κ_B (NF-_κ_B) and type I interferon (IFN) signalling, together with cellular processes such as generation of reactive oxygen species (ROS) and autophagy (Coutermarsh-Ott et al., 2016a). Particular attention is attributed to NLRX1, which attenuates inflammatory pathways in multiple infectious and autoimmune diseases. Here, we discuss the role of NLRX1 both as a traditional PRR and as a modulator of cellular homeostasis by recapitulating studies from its initial description until recent findings.

## NLRX1 Functions as a Pathogen Recognition Receptor

NLRX1, also named NOD5, NOD9, or CLR11.3, is the first and only NLR to be targeted to the mitochondria. Like all NLRs, NLRX1 contains a central nucleotide binding domain (NBD) and a C-terminal LRR domain. However, its unconventional N-terminal domain shares no apparent homology to other domains and contains a mitochondrial targeting sequence (MTS) in its first 39 amino acids (Moore et al., 2008; Tattoli et al., 2008; Arnoult et al., 2009). Initially, NLRX1 was described as a mitochondrial outer membrane (MOM)-associated protein (Moore et al., 2008). Subsequently, studies failed to detect NLRX1 at the MOM, but rather in the mitochondrial matrix. Arnoult et al. (2009) showed that the translocation of NLRX1 to the matrix required mitochondrial inner membrane potential. Once in the matrix, the N-terminal MTS is removed similarly to other mitochondria targeted proteins (Arnoult et al., 2009). Supporting its role in antiviral immunity, structural and functional analysis of NLRX1 identified a C-terminal RNA-binding element that interacts directly to ssRNA, dsRNA and poly I:C, but not DNA, probably *via* multiple positive charges on its surface and this binding was essential for ROS production (Hong et al., 2012). Although providing structural insight to potential ligand binding, it still remains unclear how binding to viral RNA (but not DNA) could occur at the mitochondrial matrix and how this binding would promote ROS production. Interestingly, several NLRs have been shown to shuttle between distinct subcellular localizations following stimulation (Coutermarsh-Ott et al., 2016a). Several groups have indeed reported a change in NLRX1 localization upon infection. Whereas in uninfected macrophages NLRX1 localizes both in the cytoplasm and at the mitochondria, Rhinovirus infection promoted relocalization of NLRX1 from the cytoplasm to the mitochondria of epithelial cells. Similarly, infection of macrophages with the fungal pathogen, *Histoplasma capsulatum* (*H. capsulatum*), promoted NLRX1 translocation to phagosomes (Huang et al., 2018). Of note, translocation of NLRX1 to phagosomes could be of great interest in the study of the innate immune response against intracellular pathogens, including bacteria and protozoan parasites, that have developed strategies to survive inside phagolysosomes.

NLRX1 was first described over 10 years ago, mostly in the context of host defense to pathogens. Moore et al. (2008) propose that NLRX1 negatively regulates mitochondrial antiviral signalling protein (MAVS)-mediated type I IFN signaling by directly interacting with MAVS through its LRR domain at the MOM. By preventing interaction between dsRNA-activated RIG-I and MAVS, NLRX1 functions as a negative regulator of antiviral immunity and depletion of NLRX1 by siRNA resulted in decreased ssRNA viral loads in HEK293T cells after Sendai virus infection (Moore et al., 2008). However, subsequent studies failed to identify a role in the regulation of MAVS-dependent signalling, but rather showed that in the mitochondrial matrix NLRX1 could potentiate ROS production in HEK293 cells upon stimulation with TNFα and poly I:C, or infection with Shigella by interacting with the ubiquinol-cytochrome c reductase core protein II (UQCRC2), a matrix-facing protein of the complex III of the mitochondrial electron transport chain (ETC) (Tattoli et al., 2008; Arnoult et al., 2009). Mitochondria are major sources of ROS that serve as a mechanism to suppress pathogen replication *via* inflammation and apoptosis. Supporting its role in modulating ROS production, NLRX1 was shown to enhance ROS production upon Chlamydial infection (Abdul-Sater et al., 2010), poly I:C stimulation or Rhinovirus infection (Unger et al., 2014) and could bind UQCRC2 in mouse embryonic fibroblasts (MEFs) (Rebsamen et al., 2011). On the other hand, loss of NLRX1 resulted in increased ROS production following *Helicobacter pylori* (Philipson et al., 2015) or *Listeria monocytogenes* (Zhang et al., 2019) infection of macrophages. In addition, the use of an N-terminal HA-tagged NLRX1 in the initial study by Moore et al. (2008) was further questioned since the addition of an N-terminal tag prevented translocation of NLRX1 to the mitochondria and overexpression of tagged-NLRX1 resulted in a cytoplasmic pool of the NLRX1 protein probably as a consequence of overwhelmed mitochondrial import (Tattoli et al., 2008; Arnoult et al., 2009; Ling et al., 2012).

Following the first description by Moore et al. (2008) and Tattoli et al. (2008), four independent groups generated mice models to further investigate the role of NLRX1 in infection (**Table 1**). Allen et al. (2011) generated a knock out (KO) mouse model by deleting the exon 4 and 5 of NLRX1 corresponding to its nucleotide binding domain (NBD). NLRX1 KO mice challenged with LPS or infected with Influenza virus showed higher inflammation and type I IFN signaling. NLRX1 attenuated MAVS-dependent RIG-I signalling in MEFs after stimulation with poly I:C, but not in bone marrow derived macrophages (BMDMs). Although it is possible that NLRX1 function is cell type specific, differences in inflammatory response between the two cell types may be due to a more potent activation of NF-_κ_B and IRF3 pathways in MEFs compared to BMDMs (Li et al., 2019). In contrast, NLRX1 attenuated NF-_κ_B signalling in both LPS or TNFα treated MEFs and BMDMs by interacting with TRAF6 ubiquitin ligase (Allen et al., 2011). Xia et al. (2011) generated a shRNA knockdown (KD) mouse model whereby mice were shown to be more susceptible to LPS-induced septic shock, but not to poly I:C treatment. In contrast, *in vitro* KD macrophages showed increased IL-6 and TNFα production after stimulation with several TLR ligands, including LPS, poly I:C and CpG DNA (Xia et al., 2011). Similarly to Allen et al. (2011); Xia et al. (2011) showed that NLRX1 inhibits TLR-induced NF-_κ_B signalling by binding through its LRR domain to TRAF6 in unstimulated cells and the IKK complex upon LPS stimulation (Xia et al., 2011). Both studies used luciferase reporter assays and HA-tagged overexpression models to investigate the mechanism of regulation of NF-_κ_B and type I IFN pathways. These methods were subsequently described as unreliable as activation of the luciferase reporter was shown to be a non-specific effect of the overexpression of the LRR domain (Ling et al., 2012; Soares et al., 2013). In agreement, Rebsamen et al. (2011) generated a third mouse model by deleting exons 1 to 4 and showed that NLRX1 did not affect MAVS-dependent antiviral signalling following intravenous injection of poly I:C or cytokine production in MEFs, or BMDMs, following poly I:C treatment or Sendai virus infection, respectively (Rebsamen et al., 2011). Similar observations were described by Soares et al. (2013) using a fourth KO mouse model having a deletion of exon 3 after intranasal challenge with Influenza A virus or intraperitoneal injection of poly I:C. NLRX1 did not affect IL-6 production or type I IFN expression neither in BMDMs, nor MEFs, infected with Sendai virus.

**Table 1:** Mouse models to study NLRX1.

Author, Year	Knock out model	Effect of NLRX1
Allen et al. (2011)	Deletion of exon 4 and 5	*Inhibitory effect on MAVS-signaling*: NLRX1 KO mice showed increased inflammation and type I IFN signaling after LPS challenge of Influenza virus (ssRNA) infection
Xia et al. (2011)	shRNA knockdown	*Inhibitory effect on NF-KB signaling*: NLRX1 KD mice were more susceptible to LPS-induced septic shock, but not to poly I:C treatment
Rebsamen et al. (2011)	Deletion of exon 1 to 4	*No effect on MAVS signaling*: no difference between WT and KO mice after Sendai virus (ssRNA) infection or i.v injection of poly I:C
Soares et al. (2013)	Deletion of exon 3	*No effect on MAVS signaling*: no difference between WT and KO mice after intranasal challenge with Influenza A virus (ssRNA) or i.p injection of poly I:C

IFN, interferon; i.v, intra-venous; KD, knockdown; KO, knock-out; LPS, lipopolysaccharide; MAVS, mitochondrial anti-viral signaling protein; NF-KB, nuclear factor kappa-light-chain-enhancer of activated B cells; WT, wild type.

In the field of innate immunity, NLRX1 has still gained a lot of attention in recent years. Several mechanisms of action have been proposed, with many contradicting previous observations. Feng et al. (2017) proposed an interesting model, where NLRX1 negatively regulated the MAVS-mediated anti-viral IRF3 pathway, but promoted early IRF1-mediated anti-viral signaling by competitively binding dsRNA and thus preventing translational shutdown of IRF1 mediated by the dsRNA-activated protein kinase PKR. Thus, the effect of NLRX1 would depend on whether the host response is driven by IRF1 or IRF3. The mechanism of action of NLRX1 has been also shown to be cell-specific. In a model of invasive pulmonary aspergillosis (IPA), Kastelberg et al. (2020) showed that loss of NLRX1 in CD103+ dendritic cells resulted in higher mortality in mice due to an increased IL-4 production *via* enhanced c-Jun N-terminal kinase (JNK) pathway. On the contrary, the loss of NLRX1 in non-hematopoietic cells resulted in increased IL-6 and CXCL8/IL-8 production *via* elevated p38 activation and was associated with increased innate immune cell recruitment and survival *in vivo* (Kastelberg et al., 2020). Many factors have also been shown to compete with NLRX1. For example, FAS-associated factor-1 (FAF1) promoted anti-viral signaling and the type I IFN response by targeting NLRX1 and preventing its binding to MAVS (Kim et al., 2017). Another study showed that NLRX1 induced the degradation of MAVS through recruitment and interaction with poly(rC) binding protein 2 (PCBP2) upon hepatitis C virus infection (Qin et al., 2017). In addition, NLRX1 inhibited type I IFN signaling also in response to DNA viruses and promoted viral replication by sequestering the cytosolic DNA sensor STING (Guo et al., 2016), or to herpesviruses by inhibiting MAVS-signaling (Ma et al., 2017). On the other hand, NLRX1 restricted virus replication by interacting with a viral non-structural protein 9 (Nsp9) RNA-dependent RNA polymerase domain of PRRSV2 virus (porcine reproductive and respiratory syndrome virus-2) (Jing et al., 2019). Taken together, a consensus regarding ligand specificity, stimuli-specific subcellular localization and mechanism of action is yet to be further defined.

## NLRX1 Attenuates Tumorigenesis and Inflammation

NLRX1 was also shown to attenuate inflammation and regulate cellular homeostasis independently of its role in pathogen recognition. The loss of NLRX1 is associated with more severe inflammation and tissue damage in different models of inflammation such as cancer. In SV40 transformed MEFs, but not in primary cells, NLRX1 mediated resistance to mitochondria dependent extrinsic apoptosis induced by TNFα-cycloheximide (CHX), but susceptibility to intrinsic apoptosis caused by glucose starvation or endoplasmic reticulum (ER) stress (Soares et al., 2014). Coherently, NLRX1 KO mice had fewer tumors in an azoxymethane induced colorectal cancer model probably due to a lower rate of intrinsic apoptosis, however, there was increased pathology in a dextran sodium sulfate (DSS)/azoxymethane colitis model in which accumulation of inflammatory mediators such as TNFα trigger excessive extrinsic apoptosis (Soares et al., 2014). Further studies confirmed the role of NLRX1 in tumorigenesis. Singh et al. (2015) showed that NLRX1 suppressed tumorigenicity and migration in human breast cancer cell lines by promoting TNFα-induced mtROS production and caspase-8 mediated apoptosis (Singh et al., 2015). Similarly, in a model of urethane-induced histiocytic sarcoma, NLRX1 attenuated cancer progression by negative regulation of pro-survival NF-_κ_B and AKT signalling pathways in BMDMs (Coutermarsh-Ott et al., 2016b). Additional mouse models including Cre-mice lacking NLRX1 in intestinal epithelial cells (Tattoli et al., 2016), or bone marrow chimeras lacking NLRX1 in non-hematopoietic cells (Koblansky et al., 2016) confirmed the role of NLRX1 as a tumor suppressor in intestinal epithelial cells. Interestingly, NLRX1 expression was decreased only in cancer cell lines with high metastasis potential and NLRX1 was shown to promote apoptosis (Kang et al., 2015) and senescence in tumor cells while repressing invasiveness by inhibition of AKT pathway (Hu et al., 2018; Singh et al., 2019).

Although NLRX1 was first described to attenuate inflammation in transformed cells, loss of NLRX1 is also linked to more severe inflammation in different models of tissue injury. The effect of NLRX1 has been studied extensively in the context of central nervous system (CNS) inflammation. In the brain, NLRX1 attenuated macrophage-induced inflammation in mice in a model of experimental encephalomyelitis (Eitas et al., 2014), and the NF-_κ_B responsive genes in a model of traumatic brain injury (Theus et al., 2017). Gharagozloo et al. (2019) later further confirmed the observations of Eitas et al. (2014) and showed that NLRX1 prevented CNS inflammation, immune cell infiltration and the generation of neurotoxic astrocytes in a model of spontaneous experimental autoimmune encephalomyelitis (Gharagozloo et al., 2019). In addition, the LRR domain of NLRX1 could alleviate autoimmune encephalomyelitis when delivered to the brain by reducing tissue inflammation, CD45+ immune cell and CD4+ IFNy+ T cell infiltration (Koo et al., 2020). Similarly to Allen et al. (2011) and Xia et al. (2011); Peng et al. (2020) propose in a model of cerebral ischemia/reperfusion injury that DJ-1 (PARK7), a potential antioxidant mitochondrial protein, promoted NLRX1 dissociation from TRAF6 to negatively regulate NF-_κ_B signalling (Peng et al., 2020). In other tissues, NLRX1 has been shown to regulate inflammation in a similar manner. Ma et al. (2019) showed that NLRX1 attenuated LPS-induced inflammation and apoptosis in chondrocytes by negative regulation of NF-_κ_B signalling in a model of osteoarthritis (Ma et al., 2019). How NLRX1 regulates NF-_κ_B signalling remains unclear. Similarly to MAVS, the targeting of cytosolic TRAF6 raises again the question of the localization of NLRX1. In addition to a potential, maybe minor, cytoplasmic pool of NLRX1, it is possible that TRAF6 translocates to the mitochondria upon stimulation. Interestingly, West et al. (2011) showed that stimulation of cell surface-bound TLRs such as TLR4 and TLR1/2 of RAW macrophages resulted in the translocation of TRAF6 at the mitochondria, but it was not the case after stimulation with TLR3 agonist, poly I:C. Thus, the effect of NLRX1 on NF-_κ_B signalling may highly be dependent on the upstream TLR stimulation.

On the other hand, protection against inflammation has also been linked to MAVS-dependent signaling. NLRX1 protected against inflammation in mice in a smoke-induced chronic obstructive pulmonary disease model by inhibiting MAVS-RIG-I pathway, or by inhibiting MAVS-dependent NLPR3 inflammasome activation in hypoxia-induced cardiomyocytes in two different models of myocardial injury (Li et al., 2016; Tong et al., 2020). Although most often linked to increased inflammation and lesion severity, the loss of NLRX1 also promoted healing by enhancing angiogenesis through miR-195 upregulation (Liu et al., 2018), or by increased expression of wound healing factors such as epidermal growth factor (EGF) and transforming growth factor beta 1 (TGFβ1) following injury (Tattoli et al., 2016).

## NLRX1 Regulates Mitochondrial Function and Metabolism

Unlike other NLRs, NLRX1 localizes at the mitochondria, the central hub for both metabolism and immunity. Recent studies suggest that NLRX1, instead of being a traditional PRR, may in fact play a more general role in the maintenance of mitochondrial physiology and function. In NLRX1 knock-in (KI) neuroblastoma cells, NLRX1 associated with GTPase dynamin-related protein 1 (DRP1), a major regulator of mitochondrial dynamics, resulted in increased mitochondrial fission, and the mitochondria in these cells showed morphological abnormalities (Imbeault et al., 2014). Similar observations were described in auditory cells in which overexpression of NLRX1 resulted in mitochondrial swelling and breakage of the cristae (Yin et al., 2018). On the other hand, loss of NLRX1 resulted in decreased mitochondrial membrane potential and in increased caspase-3 mediated mitochondrial apoptosis in virus infected BMDMs (Jaworska et al., 2014). However, the protective effect of NLRX1 was mediated by interaction with a viral protein PB1-F2 of Influenza A virus, thus not providing evidence of a more general mechanism of action.

The activity of mitochondrial respiratory chain complexes is important for the regulation of ATP levels within the cell. In addition to ATP, the mitochondrial respiratory chain is also a major source of mitochondrial ROS (mtROS). NLRX1 regulates mtROS by modulating the two major sites of mtROS production, the complex I and complex III of the ETC. NLRX1 protected against mitochondrial damage and oxidative stress in kidney epithelial cells and loss of NLRX1 resulted in increased mitochondrial fragmentation, oxidative phosphorylation (OXPHOS), and complex II/III activity (Stokman et al., 2017). Mechanistically, NLRX1 locates at the mitochondrial matrix where it associates with the Fas-activated serine-threonine kinase family protein-5 (FASTKD5), a component of mitochondrial RNA granules. This association was shown to negatively regulate RNA processing of mitochondrial complex units (Singh et al., 2018). Interestingly, NLRX1 protected against mitochondrial injury in both infection (Zhang et al., 2019), and in *in vitro* models of injury by sodium azide or glucose starvation (Chu et al., 2019). Mitochondrial injury leads to increased inflammation, and thus NLRX1 may negatively regulate inflammation by direct maintenance of mitochondrial homeostasis.

Mitochondria emerge as key players in maintenance of homeostasis and in host innate defense mechanisms. Any metabolic stress or infectious agents that alter the function or damage mitochondria will lead to the fragmentation and subsequent degradation of defective and/or depolarized mitochondria by mitophagy or by apoptosis (Toyama et al., 2016). Impaired removal of damaged mitochondria leads to the release of multiple DAMPs to the cytosol, or to the extracellular space which triggers innate immune responses and inflammation (Banoth and Cassel, 2018; Harris et al., 2018). Intriguingly, NLRX1 could regulate autophagy and/or mitophagy in infectious diseases and in models of cell stress. Autophagy is a highly conserved process requiring mitochondrial function to recycle and degrade proteins within an autophagosome-lysosome fusion mainly upon cellular stress such as starvation (Shibutani et al., 2015; Singh et al., 2019). Autophagy is also induced by many pathogens including bacteria, viruses and parasites and is a potent host defense mechanism. In addition, autophagy has been shown to suppress inflammasome activation, to negatively regulate RLR-mediated type I IFN production and to promote MHC class II antigen presentation (Shibutani et al., 2015). Some pathogens, including bacteria and viruses, can impact mitochondrial metabolism, usually by inhibiting mitochondrial OXPHOS to favor their intracellular survival. On the contrary, the metabolic modifications can promote the immune response (Tiku et al., 2020). Mitochondrial dynamics and mitophagy are important mechanisms to regulate innate immune response to infection and can mediate macrophage activation towards a pro-inflammatory phenotype and microbial clearance (Gkikas et al., 2018).

Lei et al. (2012) showed that NLRX1 inhibited type I IFN production in MEFs by associating with mitochondrial matrix protein Tu translation elongation factor (TUFM) *via* its N-terminus. TUFM interacts with the autophagy-related proteins Atg5-Atg12 and promotes autophagy during vesicular stomatitis viral infection. The same group showed TUFM-NLRX1 interaction also in cancer cells, in which NLRX1 promoted cetuximab, an epidermal growth factor receptor (EGFR) inhibitor, -induced autophagy and mediated resistance to cancer treatment (Lei et al., 2016). Interestingly, cetuximab has been shown to enhance ER-Stress- and mitochondria-mediated apoptosis through ROS production (Kang et al., 2020). Interaction with TUFM was further confirmed in macrophages infected with a fungal pathogen, *H. capsulatum*. NLRX1-TUFM promoted LC3-associated phagocytosis (LAP) of *H. capsulatum* and LAP-mediated cytokine production *via* MAPK-AP-1 pathway (Huang et al., 2018). Some pathogens have evolved to escape from autophagy-mediated killing or even use the autophagy machinery as part of their replication cycle. A bacterial pathogen, *Listeria monocytogenes* (*L. monocytogenes*) was shown to induce NLRX1-dependent mitophagy to avoid killing (Zhang et al., 2019). Mechanistically, for the first time, Zhang et al. (2019) showed that NLRX1 contains a LC3-interacting domain (LIR), and its direct interaction with LC3 induced *L. monocytogenes-*dependent mitophagy and suppressed mtROS production. Similarly, the human papilloma virus 16 (HPV16) has developed a mechanism to suppress the STING-mediated DNA sensing pathway in an autophagy-dependent manner. Luo et al. (2020) showed that the E7 protein of HPV16 could bind NLRX1 and promoted autophagy-dependent degradation of STING leading to decreased anti-viral type I IFN production (Luo et al., 2020). On the other hand, NLRX1 inhibited bacterial invasion and the consequent autophagosome formation and maturation following Streptococcus A infection in HeLa cancer cells by interacting with the Beclin-1-UVRAG complex *via* the NACHT domain, similarly to other NLRs such as NLRC4 and NLRP4 (Aikawa et al., 2018). Likewise, in other cancer cell lines, NLRX1 suppressed TNFα induced autophagy and mitophagy (Singh et al., 2019), potentially indicating that the effect of NLRX1 on autophagy might differ between transformed and primary cell lines.

With the expansion of the immunometabolism field, growing interest has been attributed to the role of NLRX1 as a modulator between inflammation and metabolism. The first observations that NLRX1 could directly regulate metabolism were made in cancer cells. These cells are highly glycolytic and inhibition of glycolysis was shown to result in decreased NLRX1 expression in both primary and transformed MEFs (Soares et al., 2014). Similarly, in other cancer cell lines, NLRX1 downregulated the mitochondrial respiratory complex I and III activity to promote the metabolic switch towards aerobic glycolysis (Singh et al., 2015; Singh et al., 2019). The effect on metabolism is likely to be cell type and microenvironment dependent. Indeed, in CD4+ T cells, NLRX1 was shown to promote mitochondrial oxidative metabolism and NLRX1 KO mice had increased populations of both Th1 and Th17 inflammatory cells. In addition to glucose metabolism, NLRX1 regulated fatty acid metabolism. Under a high fat diet, NLRX1 KO mice had increased fatty acid-dependent OXPHOS and were protected from diet-induced hepatic steatosis (Kors et al., 2018) and pancreatic lipid accumulation (Costford et al., 2018). Interestingly, different lipids such as coenzyme A-containing fatty acids and sterols were shown to bind the LRR domain of NLRX1 and punicic acid (PUA), a polyunsaturated fatty acid, exerted anti-inflammatory effects in a NLRX1-dependent manner in LPS-activated BMDMs or in a DSS-induced colitis mice model (Lu et al., 2015). In addition, NLRX1 regulated glutamine metabolism as NLRX1 KO mice showed increased glutamine utilization and interestingly glutamine supplementation alleviated the severity of inflammatory bowel disease in mice (Leber et al., 2018). Altogether, NLRX1 is a promising therapeutic target as an immunometabolic regulator of inflammation. As a proof of principle, Leber et al., 2019a developed a small molecule, NX-13, that showed anti-inflammatory effects by activating NLRX1 of a small molecule in an *in vivo* model of inflammatory bowel disease (Leber et al., 2019a; Leber et al., 2019b).

## Discussion

**Figure 1:**
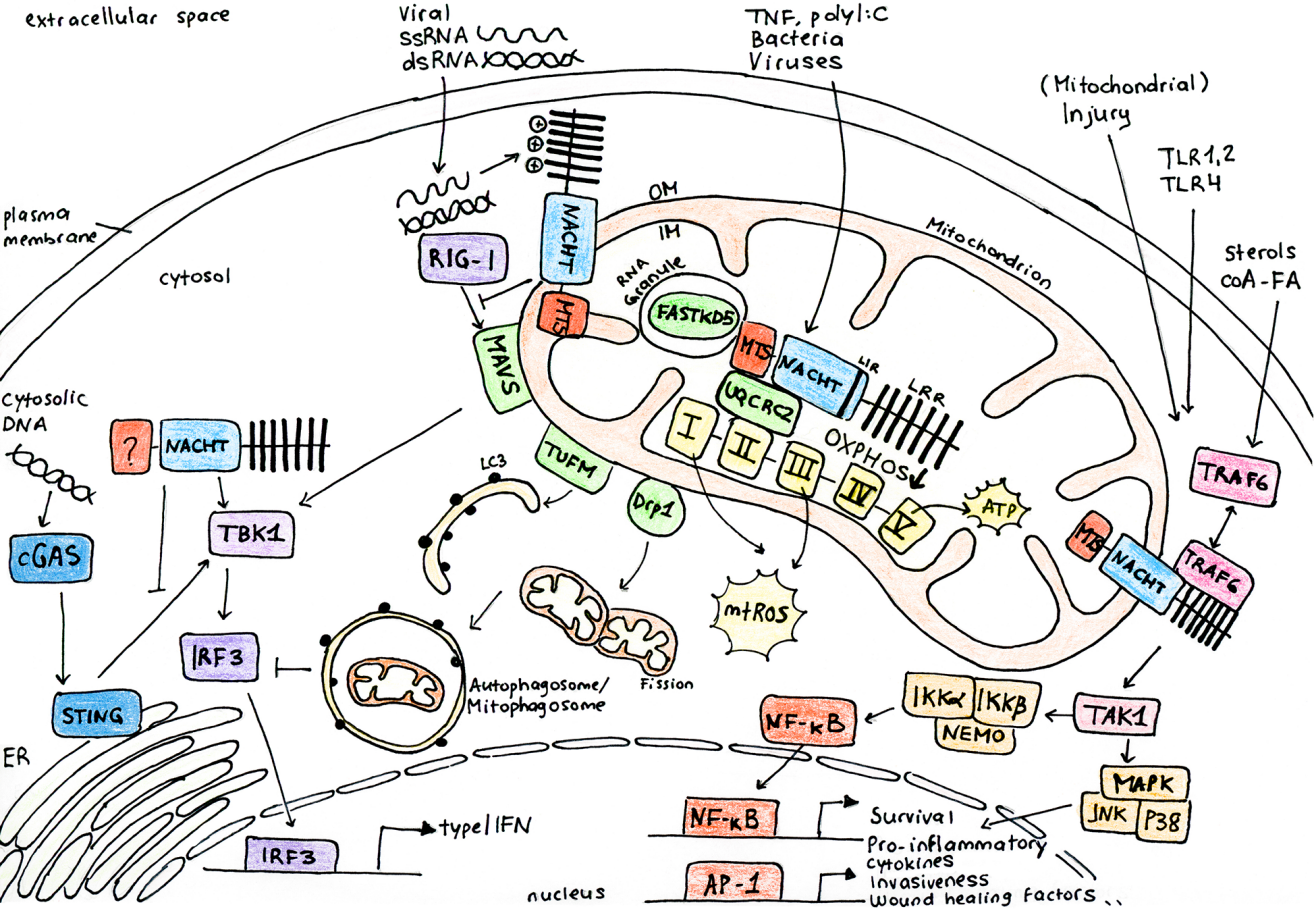
NLRX1 regulates multiple cellular pathways to control inflammation in response to infection and stress. NLRX1 is addressed to the mitochondria *via* the N-terminal mitochondrial targeting sequence (MTS). Studies identified a C-terminal RNA-binding element and coherently, NLRX1 plays a role in anti-viral signaling by inhibition of type I interferon signaling through direct interaction with the outer membrane (OM) mitochondrial anti-viral signaling protein (MAVS). However, some studies describe that NLRX1 attenuates viral replication in the cytoplasm *via* a MAVS-independent mechanism, potentially through binding of stimulator of interferon genes (STING). Inhibition of type I IFN signalling following virus infection is associated to enhanced autophagy and mitophagy through association of NLRX1 with either the Tu translation elongation factor (TUFM) or GTPase dynamin-related protein 1 (DRP1). Similarly, NLRX1 is associated to enhanced mitochondrial reactive oxygen species (mtROS) production and decreased oxygen consumption and mitochondrial oxidative phosphorylation (OXPHOS) following virus or bacterial infection through interaction with the mitochondrial protein ubiquinol-cytochrome c reductase core protein II (UQCRC2). Growing interest has been attributed to the role of NLRX1 as a modulator between inflammation and metabolism. Following cellular stress, NLRX1 inhibits activation of NF-_κ_B through its direct interaction with TRAF6 or I_κ_B kinase (IKK) complex. NLRX1 may indeed play a more general role in the maintenance of mitochondrial physiology and cellular homeostasis. AP-1, activator protein 1; cGAS, cyclic GMP-AMP synthase; ER, endoplasmic reticulum; FASTKD5, FAST kinase domain-containing protein 5; IKKa, I_κ_B kinase subunit α; IKKb, I_κ_B kinase subunit β; IM, inner membrane; IRF3, interferon regulatory factor 3; JNK, c-Jun N-terminal kinase; LC3, microtubule-associated protein 1A/1B-light chain 3; LIR, LC3-interacting domain; LRR, leucine rich repeat domain; MAPK, mitogen-activated protein kinase; NACHT, central nucleotide-binding oligomerization domain; NEMO, NF-_κ_B essential modulator; NF-_κ_B, nuclear factor kappa-light-chain-enhancer of activated B cells; p38, p38 mitogen activated protein kinase; RIG-I, retinoic acid-inducible gene I; TAK1, transforming growth factor-β activated kinase-1; TBK1, TANK-binding kinase 1; TRAF6, TNF receptor-associated factor 6.

**Figure 2:**
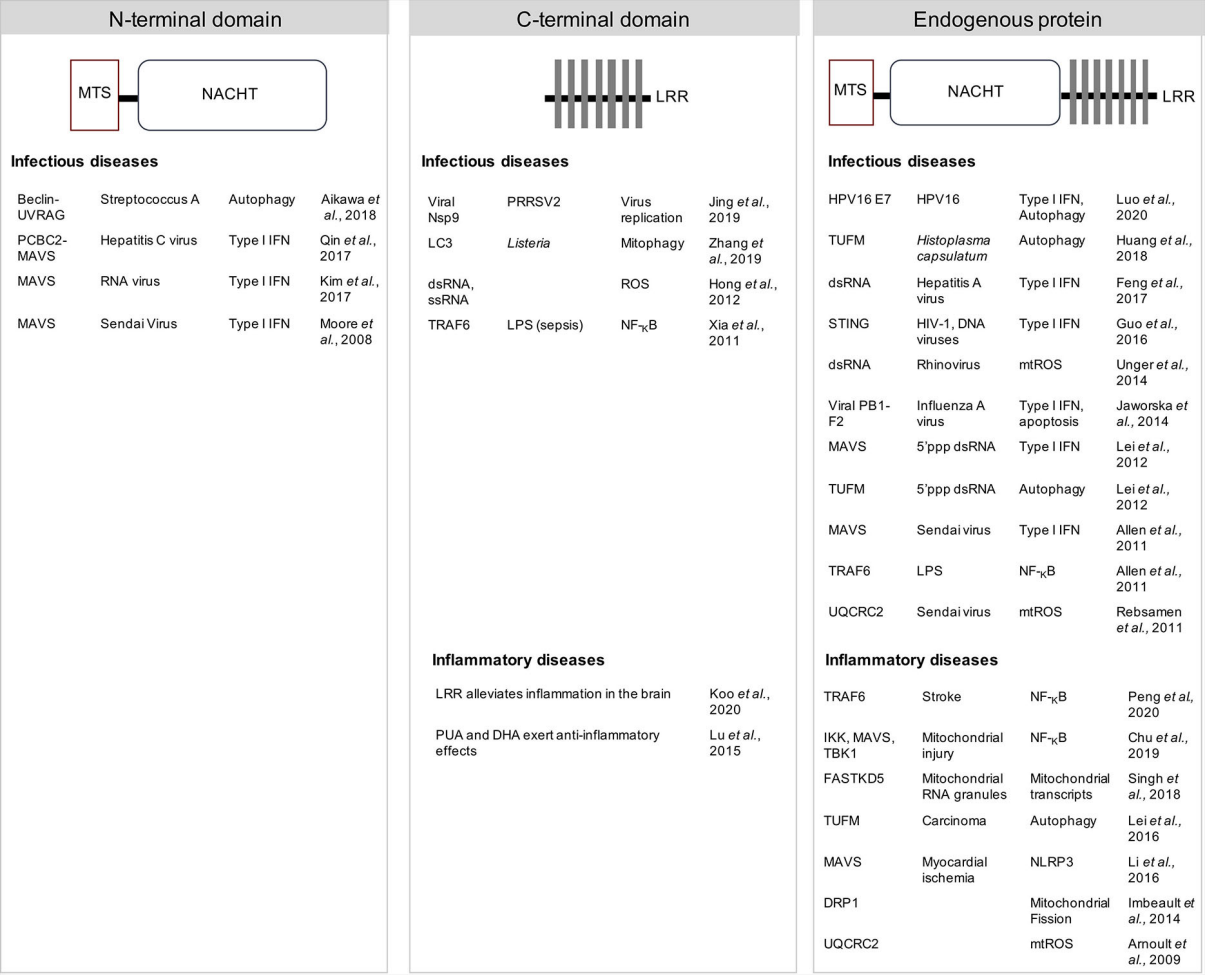
NLRX1 modulates innate immunity and inflammation. Multiple mechanisms of action and direct interacting partners are proposed in different experimental models. The N-terminal central nucleotide-binding domain (NACHT) of NLRX1 plays a role in attenuating MAVS-dependent anti-viral signalling by inhibiting the type I interferon (IFN) response against both ssRNA and dsRNA viruses. In addition, the NACHT domain was necessary to inhibit invasion and autophagosome formation upon Streptococcus A infection. Multiple functions are proposed to depend on the C-terminal leucine rich repeat (LRR). LRR was shown to directly bind a viral RNA polymerase Nsp9 of porcine reproductive and respiratory syndrome virus-2 (PRRSV2) to limit virus replication, or to bind LC3 to induce *Listeria monocytogenes*-dependent mitophagy, or to induce mtROS upon RNA stimulation. The LRR also limited inflammation in both infectious and inflammatory disease models. Many binding partners have been proposed using the endogenous protein. NLRX1 could play a role in modulating type I IFN signalling, NF-_K_B pathway, autophagy and several mitochondrial functions. Thus, a consensus regarding the exact mechanism of action of NLRX1 still requires further investigation. DRP1, GTPase dynamin-related protein 1; FASTKD5, FAST kinase domain-containing protein 5; HPV, human papillomavirus; IKK, I_κ_B kinase; LC3, microtubule-associated protein 1A/1B-light chain 3; LPS, lipopolysaccharide; MTS, mitochondrial targeting sequence; MAVS, mitochondrial anti-viral signaling protein; NF-_κ_B, nuclear factor kappa-light-chain-enhancer of activated B cells; NLRP3, NOD-, LRR- and pyrin domain-containing protein 3; Nsp9, a viral non-structural protein 9; PB1-F2, influenza virus protein PB1-F2; PCBC2, poly(rC) binding protein 2; ROS, reactive oxygen species; STING, stimulator of interferon genes; TBK1, TANK-binding kinase 1; TRAF6, TNF receptor-associated factor 6, TUFM, Tu translation elongation factor; UQCRC2, ubiquinol-cytochrome c reductase core protein II; UVRAG, UV irradiation resistance-associated gene.

NLRX1 is the only known mitochondria-associated innate immune receptor of the NLR family and it has now become evident that its role extends from traditional pathogen recognition to the regulation of different cellular functions to control inflammation (**Figure 1**). However, as highlighted by the diverse effects of NLRX1 that have been described, the exact mechanism through which NLRX1 influences inflammation, the immune response or metabolism is still under debate (**Figure 2** **)**. Many open questions remain regarding ligand specificity, cellular localization and mechanisms of action. How NLRX1 would bind viral RNA from the mitochondria remains unclear. Similarly, more evidence is required to explain the interaction with cytosolic proteins such as TRAF6 or STING, or mitochondrial outer membrane proteins such as MAVS. It is possible that the function of NLRX1 is highly dependent on the cellular environment and an altered metabolic state could explain some of the differences observed in the different infectious and cancer models. Interestingly, NLRX1 has been shown to regulate autophagy, a cellular process mediated by mitochondrial function, the metabolic state of the cell and ROS production, both in infected and cancer cells and could thus provide a more general mechanism of action. Indeed, autophagy plays an important role in the immune response against intracellular pathogens as well as in the maintenance of cellular homeostasis, but it can also be used as an immune evasion strategy by pathogens that replicate within the phagolysosome. However, whether NLRX1 directly regulates inflammation *via* autophagy still requires further investigation. Taken together, as a mitochondrial protein, NLRX1 provides a direct link between innate immunity, metabolism and mitochondrial control of inflammation. Additional studies are required to further characterize this peculiar NLR, however it seems that NLRX1 may represent an interesting target as a regulator of both innate immunity and cellular physiology.

## Author Contributions

TS contributed to the literature search and wrote the first draft of the manuscript. TS and NF contributed to manuscript revision and discussion. All authors contributed to the article and approved the submitted version.

## Funding

This work was supported by the Swiss National Fund for Research (FNRS No. 310030_173180 and IZRJZ3_164176/1).

## Conflict of Interest

The authors declare that the research was conducted in the absence of any commercial or financial relationships that could be construed as a potential conflict of interest.
